# Supplementary material for: Causal Relationship between Aging and Anorexia Nervosa: A White-Matter-Microstructure-Mediated Mendelian Randomization Analysis
Source: Biomedicines. 2024 Aug 16;12(8):1874. doi: 10.3390/biomedicines12081874 (PMC11351342; doi:10.3390/biomedicines12081874)
Supplement: Supplementary file 1 [file biomedicines-12-01874-s001.zip › Supplementary Figure/Figure S4/Singe SNP plot of MR analysis for TL to ubm-b-2052.pdf]

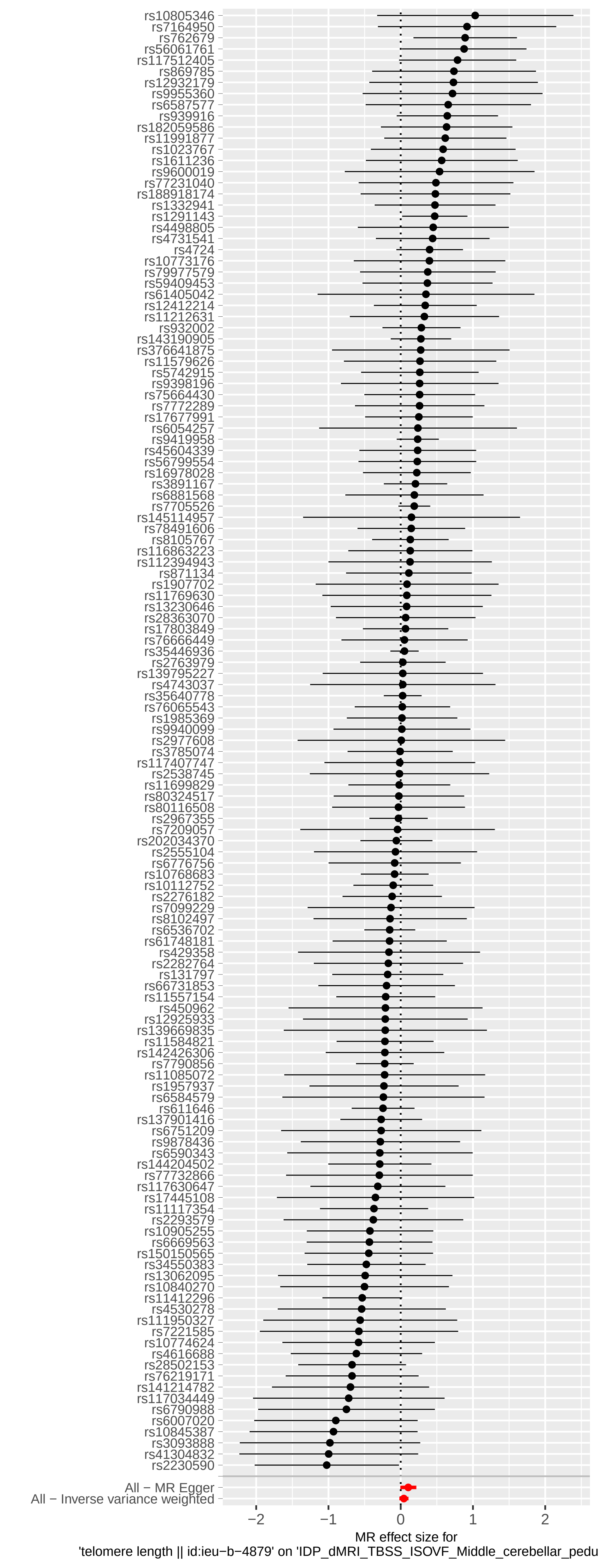
rs10805346
rs7164950
rs762679
rs56061761
rs117512405
rs869785
rs12932179
rs9955360
rs6587577
rs939916
rs182059586
rs11991877
rs1023767
rs1611236
rs9600019
rs77231040
rs188918174
rs1332941
rs1291143
rs4498805
rs4731541
rs4724
rs10773176
rs79977579
rs59409453
rs61405042
rs12412214
rs11212631
rs932002
rs143190905
rs376641875
rs11579626
rs5742915
rs9398196
rs75664430
rs7772289
rs17677991
rs6054257
rs9419958
rs45604339
rs56799554
rs16978028
rs3891167
rs6881568
rs7705526
rs145114957
rs78491606
rs8105767
rs116863223
rs112394943
rs871134
rs1907702
rs11769630
rs13230646
rs28363070
rs17803849
rs76666449
rs35446936
rs2763979
rs139795227
rs4743037
rs35640778
rs76065543
rs1985369
rs9940099
rs2977608
rs3785074
rs117407747
rs2538745
rs11699829
rs80324517
rs80116508
rs2967355
rs7209057
rs202034370
rs2555104
rs6776756
rs10768683
rs10112752
rs2276182
rs7099229
rs8102497
rs6536702
rs61748181
rs429358
rs2282764
rs131797
rs66731853
rs11557154
rs450962
rs12925933
rs139669835
rs11584821
rs142426306
rs7790856
rs11085072
rs1957937
rs6584579
rs611646
rs137901416
rs6751209
rs9878436
rs6590343
rs144204502
rs77732866
rs117630647
rs17445108
rs11117354
rs2293579
rs10905255
rs6669563
rs150150565
rs34550383
rs13062095
rs10840270
rs11412296
rs4530278
rs111950327
rs7221585
rs10774624
rs4616688
rs28502153
rs76219171
rs141214782
rs117034449
rs6790988
rs6007020
rs10845387
rs3093888
rs41304832
rs2230590
All − MR Egger
All − Inverse variance weighted
−2
−1
0
1
2
MR effect size for
'telomere length || id:ieu−b−4879' on 'IDP_dMRI_TBSS_ISOVF_Middle_cerebellar_pedu
